# Microenvironment M1/M2 macrophages and tumoral progression vary within C57BL/6 mice from same substrain in prostate cancer model

**DOI:** 10.1038/s41598-024-65960-y

**Published:** 2024-07-02

**Authors:** P. Hernández-Peralta, R. Chacón-Salinas, M. I. Gracia-Mora, G. Soldevila, J. Moreno-Rodríguez, L. Cobos-Marín

**Affiliations:** 1 Department of Microbiology and Immunology, Faculty of Veterinary Medicine and Zootechnics, Universidad Nacional Autónoma de México (UNAM), Circuito Exterior sn, 04510 Mexico City, Mexico; 2 Department of Immunology, National School of Biological Sciences, Instituto Politécnico Nacional (ENCB-IPN), 11340 Mexico City, Mexico; 3 Department of Inorganic and Nuclear Chemistry, Faculty of Chemistry, Universidad Nacional Autónoma de México (UNAM), Investigación Científica 70, 04510 Mexico City, Mexico; 4 Department of Immunology, Biomedical Research Institute, Universidad Nacional Autónoma de México, 04510 Mexico City, Mexico; 5 Research Division, Hospital Juárez de México, 07760 Mexico City, Mexico

**Keywords:** Cancer, Immunology, Diseases, Medical research, Oncology

## Abstract

Cancer mice models are critical for immune-oncology research; they provide conditions to explore tumor immunoenviroment aiming to advance knowledge and treatment development. Often, research groups breed their own mice colonies. To assess the effect of C57BL/6 mice breeding nuclei in prostate cancer development and intratumoral macrophage populations, an isotransplantation experiment was performed. C57BL/6J mice from two breeding nuclei (nA and nB) were employed for prostate adenocarcinoma TRAMP-C1 cell implantation; tumor growth period and intratumoral macrophage profile were measured. BL/6nB mice (54%) showed tumor implantation after 69-day growth period while BL/6nA implantation reached 100% across tumor growth period (28 days). No difference in total macrophage populations was observed between groups within several tumoral regions; significantly higher M2 macrophage profile was observed in tumor microenvironments from both mice groups. Nevertheless, BL/6nB tumors showed around twice the population of M1 profile (11–27%) than BL6nA (4–15%) and less non-polarized macrophages. The M1:M2 average ratio was 1:8 for group A and 1:4 for B. Our results demonstrate different tumor progression and intratumoral macrophage populations among mice from the same substrain. Data obtained in this study shows the relevance of animal source renewal for better control of murine cancer model variables.

## Introduction

Cancer is one of the most relevant challenges in modern public health and a priority in world research topics. Mortality rates have increased in time, going from 7.6 million deaths in 2008 to 8.2 in 2012^1^; 9.6 in 2018^2^; and 9.9 in 2020^3^. Cancer incidence is projected to increase by 49.7% and mortality, by 62.5% over the next 20 years^2^.

In cancer, cells acquire advantageous characteristics called hallmarks, such as maintenance of the enhanced proliferation, evasion of growth suppressors, resistance to cell death, activation of replicative immortality, and evasion of immune response, among others^4,5^. It has been shown that the acquisition of these characteristics is related to the interaction between cancer cells and other non-transformed ones, such as mesenchymal^6^ and endothelial cells^7^, adipocytes^6,8,9^, fibroblasts^10,11^, and importantly, immune system cells. All of them, but specially the last, play a central role in hampering the neoplastic process^12–14^. This set of heterotypic relationships located within the tumor conform the tumor immune microenvironment (TIME)^14–16^.

One of the most relevant cells of TIME establishment are macrophages due to their phagocytic capacity, antigen presentation, cytokine production, and regulation of pro- and anti-inflammatory responses^17,18^. Macrophages participate in all phases of the neoplastic process and, because of their distribution, they are called tumor-associated macrophages (TAMs). Macrophages act in the immunosurveillance of neoplasms through their phagocytic and oxidative activity as well as their progression since they provide signals for cell growth, extracellular matrix remodeling, angiogenesis, intravasation of tumor cells, invasion, and metastasis^19–23^. In many types of cancer, macrophage populations are equal or higher than neoplastic cells^20^. There are two predominant macrophage profiles: inflammatory or classically activated M1, characterized by TLRs activation, production of IL-1, IL-6, TNFα, IL-12, and high amounts of nitric oxide through the enzyme nitric oxide synthase 2 (NOS2 or iNOS); and M2 or alternatively activated macrophages, induced by IL-4, IL-13, producers of IL-10, transforming growth factor beta (TGFβ), and characterized by the expression of CD206, CD220 receptors on its surface, as well as the presence of the intracellular enzyme arginase^18,21,24^. Macrophages are key to the biology of tumor development, whereby they have been proposed as the target of new therapies to modify TIME and restrain tumor development^17,23^. To advance the knowledge of TIME, it is essential to have reliable experimental animal models.

The mouse (*Mus musculus musculus*) has metabolic and immunological systems similar to those of the human being^25^ and is the most used species in cancer development models that allow the study of TIME and other cancer events as metastasis^26^. Among the murine models of cancer study are those induced environmentally through radiation, chemicals, and viral and bacterial infections^27–32^, as well as transgenic animals capable of developing different types of neoplasms^32,33^. Routinely used models include xenotransplantation, in which athymic immunodeficient mice are able to accept human tumor cells and develop the corresponding neoplasia^32^ and isotransplantation, which consists in the implantation of murine origin tumor cells with shared haplotype to the recipient mouse strain^34,35^. Among murine strains, C57BL/6 mice are the best known inbred strain worldwide and are extensively used in the study of the immune system and cancer^26^. The competence of mice to be employed in cancer models is central in cancer immunology research. Despite careful reproductive management by the producing companies^32,36^, a wide range of genotypic and phenotypic differences have been described within the BL/6 strain^33^.

The aim of the present work was to assess the differences in the tumor development process and the macrophage profile in TIME among isotransplanted C57BL/6 mice of different breeding nuclei from the same animal facility.

## Results

### Mice from group B showed less tumor appearance and tumor growth was slower compared to group A

**Figure 1:**
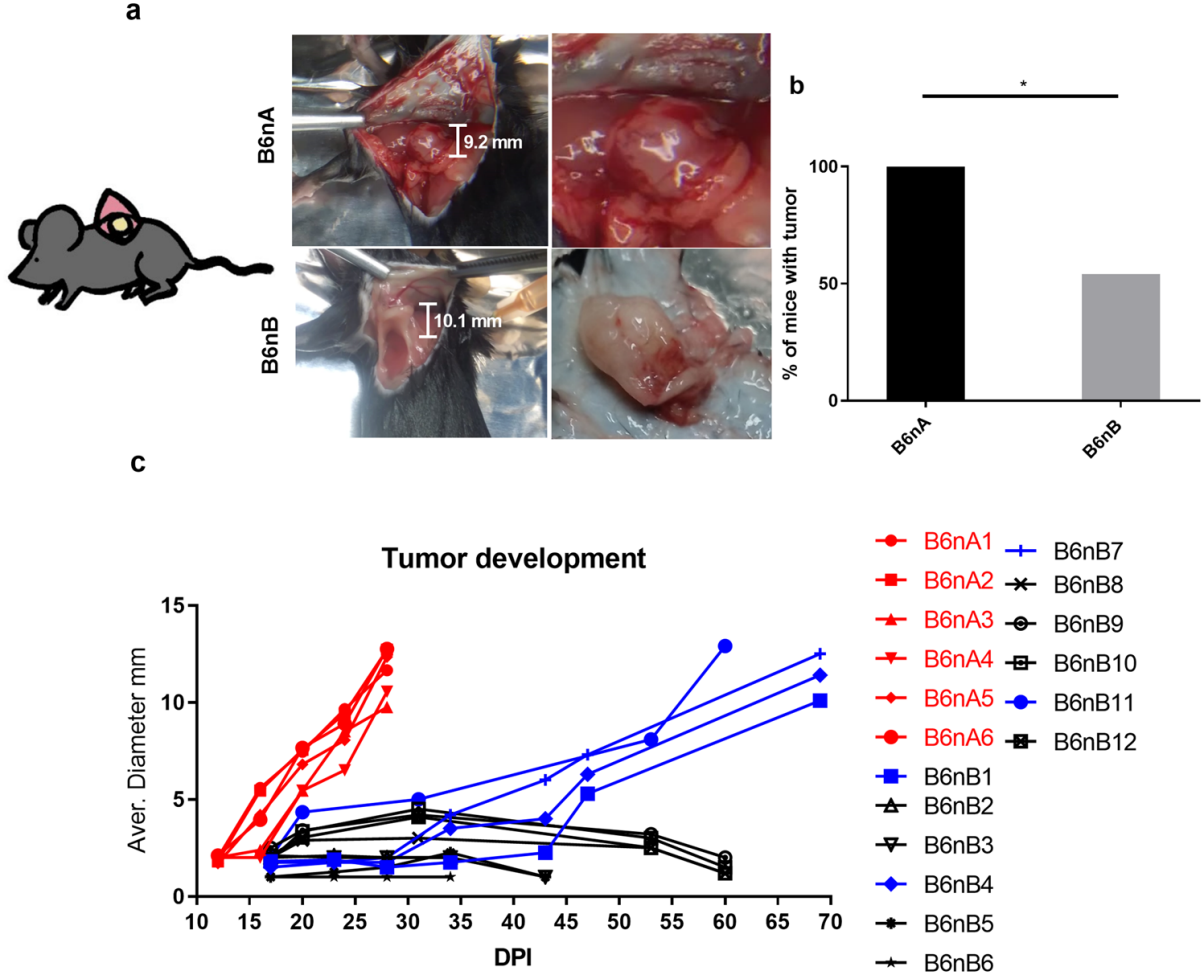
Implantation and tumor growth of TRAMP-C1 cells in C57BL/6 mice from two breeding nuclei. (**a**) Scheme of tumor location at interescapular region on mice, representative image of the tumors obtained at day 28 (BL/6nA) and 70 (BL/6nB). (**b**) Percentage of mice with successful tumor implantation (*P* = 0.03) (**c**) Tumor growth dynamics of implanted tumors by evaluation of mean tumor diameter; in red BL/6 mice from nucleus A, in blue mice from nucleus B with successful implantation, in black mice from nucleus B with tumor remission after implantation.

In the BL/6nA group, appearance of tumors at the inoculation site occurred at day 12 post-implantation (PI) with a success rate of 100% (6/6), while tumors in BL/6nB mice were observed at day 17 PI in 54% of the animals (12/22) (Chi-Square test, α = 0.05, *P* = 0.03). All tumors had an ellipsoidal shape (Fig. 1a and b). Tumors implanted in BL/6nA mice showed homogeneous growth and reached their maximum size at day 28 PI. From 12 tumors implanted in BL/6nB mice, 4 reached their maximum size in a period of 60–69 days PI and 8 regressed between days 35 and 60 PI (Fig. 1c), no differences in average weight between groups was observed (Tukey test, α = 0.05, *P* = 0.81) data not shown.

### The percentage of total macrophages in the tumor microimmunoenvironment shows no differences between groups

**Figure 2:**
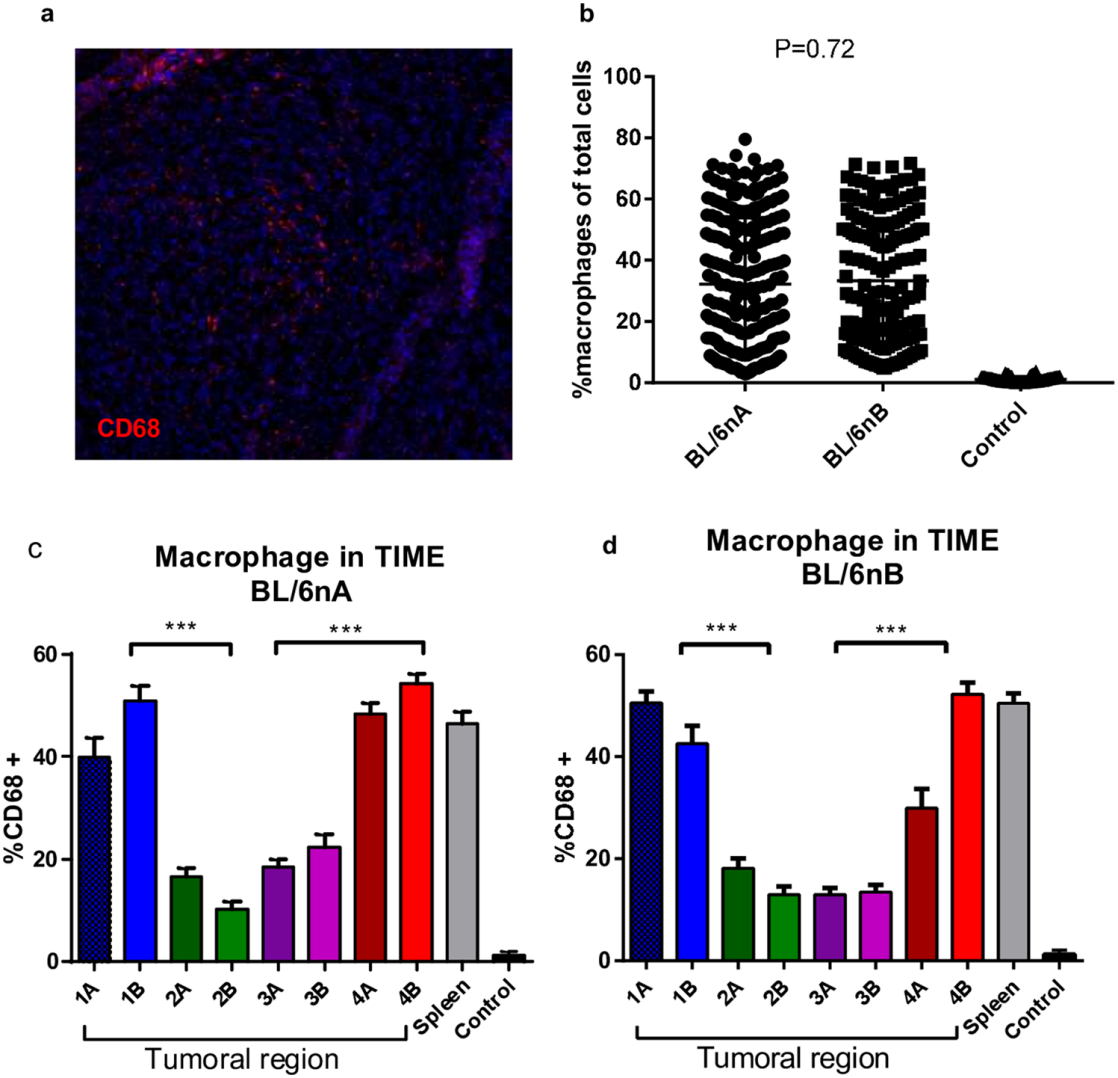
Detection of CD68 + cells within the TIM(E) (**a**) Representative image of tissue sections labeled anti-CD68 for macrophages. (**b**) Comparison between total macrophages among the tumors obtained from the BL/6nA group compared to those obtained from the BL/6nB group. (**c**) Comparison between macrophages populations present in each tumor regions for the BL/6nA and BL/6nB groups. Spleen. Macrophage staining control in spleen cut. ***P < 0.001.

The presence of macrophages within TIME was evaluated when tumor size reached 9–13 mm, at day 28 in the BL/6nA group and at days 60–69 PI in the BL/6nB group; it was measured by metaXpress image analyzer for cell-by-cell identification (Fig. 2a). No differences were observed in total macrophage percentage (CD68 +) of total cells between groups (Tukey test, α = 0.05, *P* = 0.72) (Fig. 2b).

### Total population of macrophages changes according to the tumor region in both groups

We found that the concentration of macrophages changed between tumor regions. There was a significant difference (Tukey test, α = 0.05, *P* < 0.001) between total cell macrophages in the upper and lower regions (1 and 4), averaging values between 50 and 52%, when compared to the central regions (2 and 3), which ranged between 12 and 20% (Fig. 2c and d).

### M1 and M2 phenotype macrophage populations are different depending on the tumoral region

**Figure 3:**
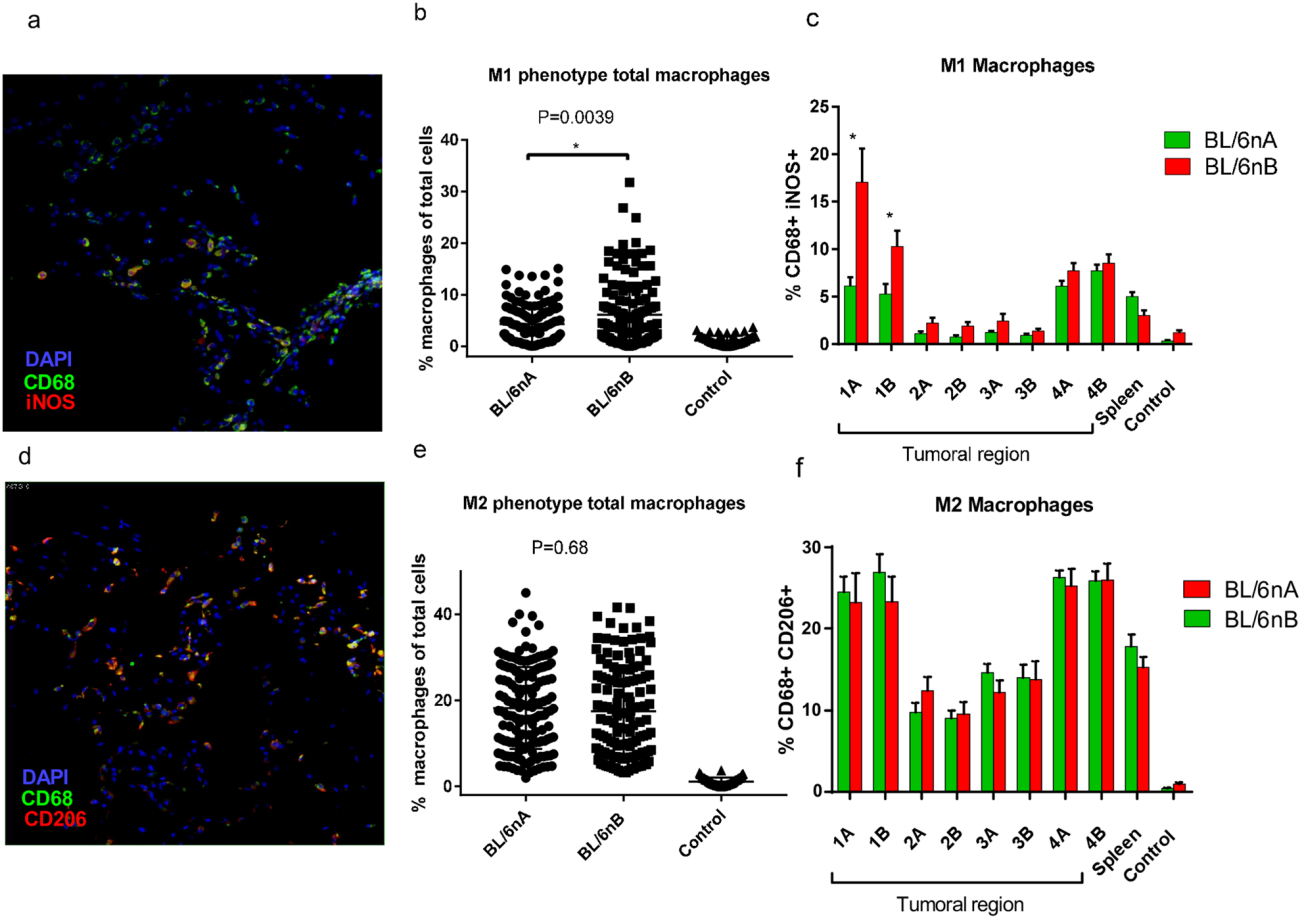
Quantification of M1 CD68 + iNOS + and M2 CD68 + CD206 + macrophages in murine prostate cancer tumor sections. (**a**) M1 DAPI + CD68 + iNOS + macrophage staining in prostate cancer tumor sections. (**b**) Comparison of total intratumoral M1 macrophages between mice from BL/6nA and BL/6nB nuclei *P = 0.0039. (c) Comparison of M1 macrophage populations between study regions for each group *P < 0.001. (**d**) M2 DAPI + CD68 + CD206 + macrophage staining in murine prostate cancer tumor sections (**e**) Comparison of total intratumoral M2 macrophages between mice from BL/6nA and BL/6nB nuclei. (**f**) Comparison of M2 macrophage subpopulations between study regions for each group *P < 0.001. Spleen. Macrophage staining control in spleen slice.

M1 phenotype macrophages were determined by the expression of CD68 + iNOS + (Fig. 3a), while the M2 phenotype was detected through the expression of CD68 + CD206 + (Fig. 3d). In tumors from group BL/6nB, a higher concentration of phenotype M1 macrophages was observed (Fig. 3b) (Tukey test, α = 0.05, *P* = 0.0039), with a marked difference in tumor regions 1A and 1B, (Fig. 3c) (Tukey test, α = 0.05, *P* < 0.001); M2 phenotype did not show differences between groups (Figs. 3e and 3f) (Tukey test, α = 0.05, *P* = 0.68). In both groups, a difference was observed in the distribution of the subpopulations depending on the tumor region, finding a greater population of the total cell in tumor regions 1 and 4, (from 5.2% ±  1.06 SEM to 17% ± 3 for M1 and from 24.5% ± 1.8 SEM to 26.9% ± 2.2 SEM for M2) as compared to central regions 2 and 3 (0.7% ± 0.18 SEM to 2.4% ± 0.7 SEM for M1 and 9.02% ± 0.9 SEM to 14.6% ± 1.0 SEM for M2) (Fig. 3c and 3f).

### Population of M1, M2, and unpolarized macrophages changes between tumoral regions

**Figure 4:**
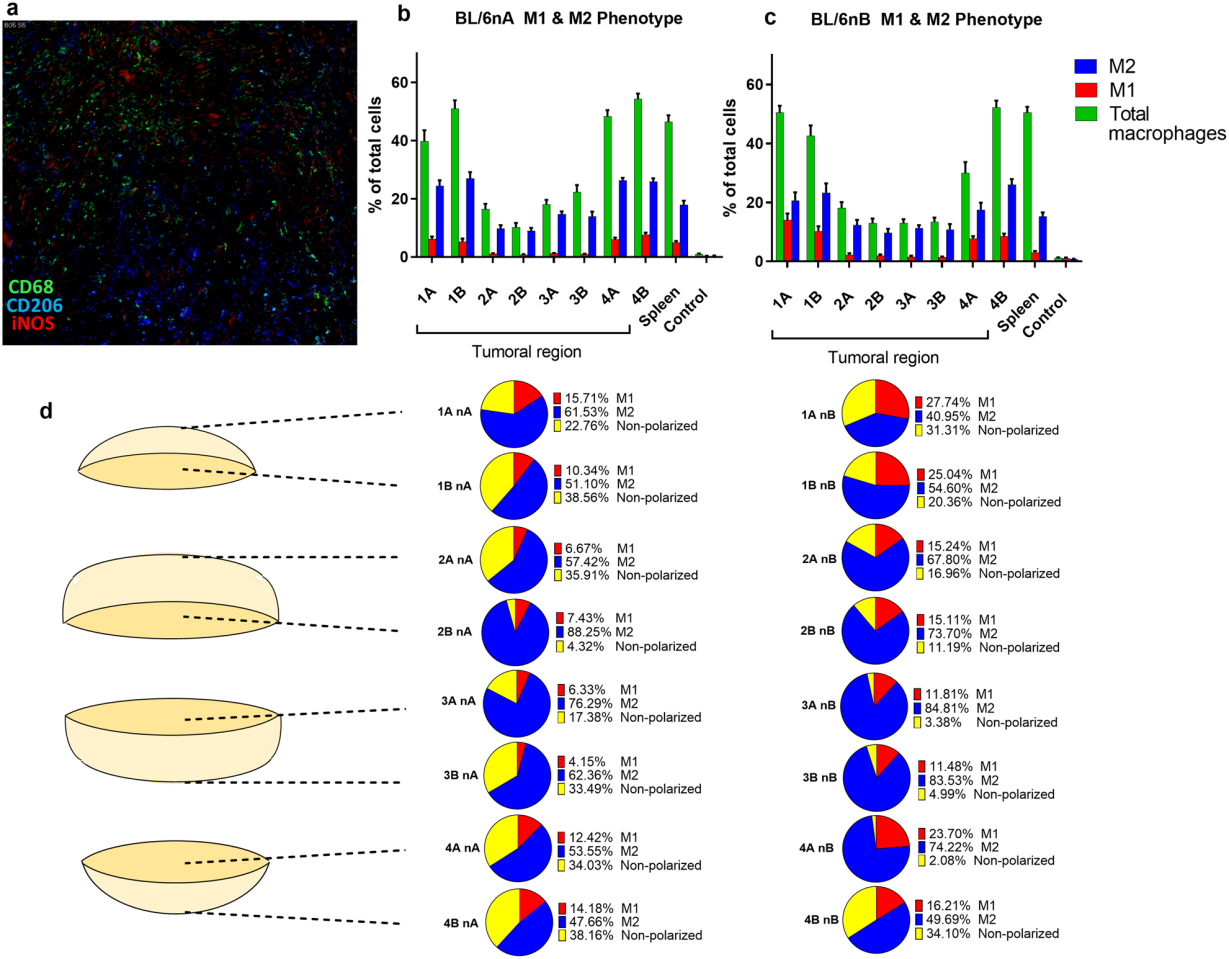
M1, M2 and non-polarized macrophages population in the different tumor regions. (**a**) Representative image of the immunostaining for the detection of the M1, M2 phenotypes and non-polarized macrophages performed on the tumors obtained from the BL/6nA and BL/6nB groups. (**b**) Quantification of M1 phenotype macrophages (red), M2 (blue) and total macrophages (green) for each of the tumor regions studied in the BL/6nA group. (**c**) Quantification of M1 phenotype macrophages (red), M2 (blue) and total macrophages (green) for each of the tumor regions studied in the BL/6nB group. (**d**) Average percentage of M1 phenotype macrophages, CD68 + iNOS (red), M2 phenotype, CD68 + CD206 + (blue) and non-polarized macrophages CD68 + iNOS-CD206—(yellow) for each of the regions studied.

To assess the total macrophage subpopulations, triple CD68, CD206, and iNOS staining was performed (Fig. 4a). In both groups, M2 phenotype macrophages presented a higher average percentage in the central regions (2 and 3), while lower values were observed in the upper and lower regions (1 and 4) (Fig. 4b and c). However, an increase in the proportion was found in the central regions 3A, 3B, and 4A of the BL/6nB group, reaching values from 74% ± 1.8 SEM to 84% ± 2.3 SEM of total macrophages (Fig. 4d). M1 macrophages presented a higher concentration in BL/6nB animals. While the percentages in the central regions (2B, 3A and 3B) of the BL/6nA group reached values between 4.15% ± 0.14 SEM and 7.43% ± 0.18 SEM, those in the mice of the BL/6nB group were between 11.48% ± 0.2 SEM and 15.24% ± 0.5 SEM. Likewise, regions 1A, 1B, and 2A in the BL/6nA group showed percentages of 15.71% ± 0.9 SEM, 10.34% ± 1.0 SEM, and 6.67% ± 0.2 SEM, respectively, which contrast with 27.74% ± 2.1 SEM, 25.04% ± 1.6 SEM, and 15.24% ± 0.5 SEM in their counterparts of group BL/6nB (Fig. 4d). The total average M1:M2 ratio was 1:8.04 (M1:M2) for the BL/6nA group and 1:4.2 for BL/6nB, and the difference between means was 3.84 ± 1.75 SEM (t-student test, α = 0.05, *P* = 0.046). The population of non-polarized macrophages (CD68 + CD206-iNOS-) was also determined for each of the tumor regions. In the central regions 3A, 3B, and 4A of the BL/6nA group, the percentages were 17.38% ± 1.54 SEM, 33.49% ± 2.41 SEM, and 34.03% ± 2.10 SEM, respectively, *vs* 3.38% ± 0.82 SEM, 4.99% ± 1.92 SEM, and 2.08% ± 0.25 SEM in the same regions of the BL/6nB group (Fig. 4d).

## Discussion

This study presents differences in the process of implantation and tumor growth between two groups of C57BL/6 mice. Animals from BL/6nB breeding nuclei showed a less successful neoplastic isotransplantation procedure, which resulted in a lower tumor implantation among 54% of the mice, a slower tumor growth period of 60–69 days, and tumor remission in some mice. In contrast, all animals from the BL/6nA group achieved a homogeneous tumor growth which reached its largest size in a shorter time (28 days). The differences between groups (*P* = 0.03) can be attributed to modifications in the genetic background of the mice. This situation has been previously described when the implantation of TRAMP cells takes place in mice from different strains, considering time of neoplastic development, and target tissues of metastasis^37–39^. About 932 genes have shown differential expression depending on the mice genetic background in prostate cancer models, many of them related to neoplastic development^39^.

No difference was observed in total macrophage concentrations between experimental groups (*P* = 0.72); nevertheless, a difference in total macrophage populations between tumor regions of both groups was found. The central ones showed total values from 15 to 20%, while in the upper and lower regions, corresponding to those close to the skin or the thorax, respectively, 30–57% were observed. The information obtained from these latter regions coincides with what has been described in various studies indicating a high proportion of macrophages that make up the tumor mass^40^, in which even higher concentrations of macrophages have been found compared to tumor cells^17,23,41^.

Abundant macrophages in TIME have been related with a poor prognosis in 80% of the cases^42^ due to its proinflammatory activity. These cells provide cytokines and reactive oxygen species into the TIME, which induces genomic instability useful to neoplastic cells for the acquisition of characteristics and abilities that allow tumor progression^4,21^. In addition, cytokines and protease synthesis contribute to the migration and intravasation of neoplastic cells in the metastasis process^43^.

We found a similar pattern in M1 and M2 subpopulations: higher concentration of both populations in the upper and lower regions compared to the central ones. However, the M1 phenotype presented significantly lower populations (from 4 to 27%) than M2 (40–84%). This correlates with the immunosuppressive activity induced by M2 macrophages in advanced stages of the neoplastic process and immunomodulation exerted by tumor cells and tumor-associated stromal cells through cytokines as TGF-β and IL-10 as well as surface receptors like PD-L1^19,41,44^. The dominance of the M2 profile translates into a decrease in the M1 profile that produces IFN-γ, nitric oxide, IL-12, and IL-18^45^.

Mice from the BL/6nB group presented around twice the amount of M1 macrophages, a reduction in non-polarized macrophages (CD68 + iNOS-CD206-) in all tumoral regions, and a total average M1/M2 ratio difference. Meanwhile, BL/6nA showed 1:8.04 (M1:M2), and BL/6nB presented 1:4.2. The possible differentiation of non-polarized macrophage towards the M1 profile remains an issue to address in further works. The higher concentration of M1 macrophages observed in this group is likely related to tumor remission and tumor growth delay found in BL/6nB mice during the development of the model. There were no significant differences in total M2 macrophages from both groups.

The aim of this study was to assess the polarized M1 and M2 populations and their spatial distribution within tumor microenvironment, two parameters proven to be related to illness severity, prognosis, and treatment effectiveness^14,46–49^. To identify the above, multiplex immunofluorescence detection devices for tissue samples, as used in the present research, are accurate data sources for tumor microenvironment cellular composition^50–52^.

The animals used in the present study come from breeding nuclei under hygienic conditions and high genetic quality management. Despite the similar origin of the groups, changes in tumor implantation and progression were observed, along with differences in one of the most relevant cell populations within TIME. During the experiment, no variations in body condition were observed between groups. However, the BL/6nB group exhibited heightened defensiveness behavior during handling. While sub-strain generation modifies mice metabolism, multiple other alterations derived from breeding have been documented, such as abnormalities in glucose and glucocorticoid pathways^53–55^, changes in locomotion, memory and anxiety^56,57^, fertility^58^, male:female ratio^58,59^, vision^60^, and amphetamine and cocaine susceptibility^61^, among others. Furthermore, there are reports on variations in genetic background associated to changes in immunological activity reflected in memory CD8 + T lymphocytes, B lymphocytes, NKTi cells, and plasmacytoid dendritic cell populations^62,63^. Due to the relevance of immune cells in TIME, population changes may have an impact on the phenotype and development of murine models on cancer research. It still remains open for future research to carry out studies to find out which changes specifically may affect tumor progression.

There are multiple research centers all around the world where mice genetic background is unknown. Researchers frequently do not know or do not care about substrains present in the animal facilities, even in research done using transgenic models that require strict protocols^64,65^. To generate stable and repeatable cancer study models, it is essential to monitor the variations between generations and reproduction nuclei. This supports the need for performing constant replenishment of mice colonies from breeding stock producing houses with genetic stability programs.

The current work studied whether breeding nuclei from C57BL/6J substrain affects the mice prostate cancer model development and macrophage populations in the tumor microenvironment. No differences were observed in macrophage total numbers nor distribution in tumoral regions. Nevertheless, C57BL/6 mice from nuclei B presented a delayed tumor development, tumor remission in 46% of the mice and significantly higher amounts of M1 profile macrophages within the tumor microenvironment. Although additional studies are required to understand the genetic changes behind these phenotypic differences, improving breeding control in mice broodstock is necessary, particularly among researchers who breed their own experimental mice and may obtain unexpected results in cancer models.

## Methods

### Animal care and management

Twenty-eight 6–8 week-old specific pathogen-free C57BL/6J (Jackson) male mice were bred and provided by the animal facility of the Biomedical Research Institute (Instituto de Investigaciones Biomedicas, IIB, UNAM, Mexico): 6 mice from breeding nuclei A (BL/6nA) and 22 from nuclei B (BL/6nB). To obtain a sufficient number of tumors from nuclei B mice, it was necessary to increase the number of implanted animals to 22, as determined by a power analysis study for statistical comparison between groups. This decision was based on a pilot study where 3 out of 6 animals implanted in the BL/6nB group developed detectable tumors. Among these, two showed tumor regression by day 36–40, while one developed a fully grown tumor (data not shown), resulting in a tumor implantation success rate of 1 in 6 implanted mice in the B group. Based on this observed occurrence rate, we calculated and adjusted the number of implanted mice required to yield 2–4 tumors for microenvironment assessment.

The animals were maintained at the animal facility of Faculty of Veterinary Medicine and Zootechnics, housed individually in polysulfone cages under standard temperature (22 °C ± 2 °C) and humidity (45% relative humidity) conditions, according to ethics guidelines. Wood shaving beds and carton cylinder enrichment were used in every cage. A 12-h light/dark cycle was set. Mice had access to food pellets and filtered water ad libitum, and handling was performed after an adaptation period of 3 days. This study was approved by the Institutional Sub-Committee for the Care and Use of Experimental Animals (SICUAE) of the Faculty of Veterinary Medicine and Zootechnics, UNAM, protocol number SICUAE DC-2019/2-4, according to the national standard (NOM-062-ZOO-1999). All methods are reported in accordance with ARRIVE guidelines.

### Cell culture

TRAMP-C1 cell line (ATCC® CRL 2730) of murine prostate epithelial adenocarcinoma was used and cultured in DMEM (Gibbco®) with 4 mM L-glutamine, 1.5 g/L sodium bicarbonate, and 4.5 g/L glucose, and supplemented with 0.005 mg/ml bovine insulin (Sigma-Aldrich), 10 nM dehydroisoandrosterone (Sigma-Aldrich), 5% fetal calf serum (Gibbco®), and 5% Nu-serum IV Corning®; it was then incubated at 37 °C with 5% CO2. At 90% of confluence, cells were detached with 3 ml 0.5% trypsin and 0.5 mM EDTA, centrifuged at 200 g, and washed twice with PBS at pH 7.4. Quantification was done with a hemocytometer starting with a 1:1 dilution of cell suspension and 0.4% trypan blue (Gibbco®).

### Animal experiment for syngeneic tumor model

3 × 10^6^ TRAMP-C1 (ATCC® CRL-2730) cells from fifth passage were implanted in a total volume of 100 µl isotonic NaCl solution. The animals were restrained in ventral decubitus and the inoculum was administered subcutaneously in the interescapular region. The day of cell inoculation was considered as day zero of the experiment. Daily measurements of tumor at the inoculation site were performed with a vernier; after 3-mm detection of the tumor mass, the average diameter was recorded. Sacrifice was carried out in accordance with animal welfare criteria, establishing the end point based on tumor size, reaching an average diameter of 9–13 mm or 20% body weight loss, hypothermia, weakness or ataxia, spontaneous behavior, and response to stimuli^32,66^.

### Sample collection and processing

**Figure 5:**
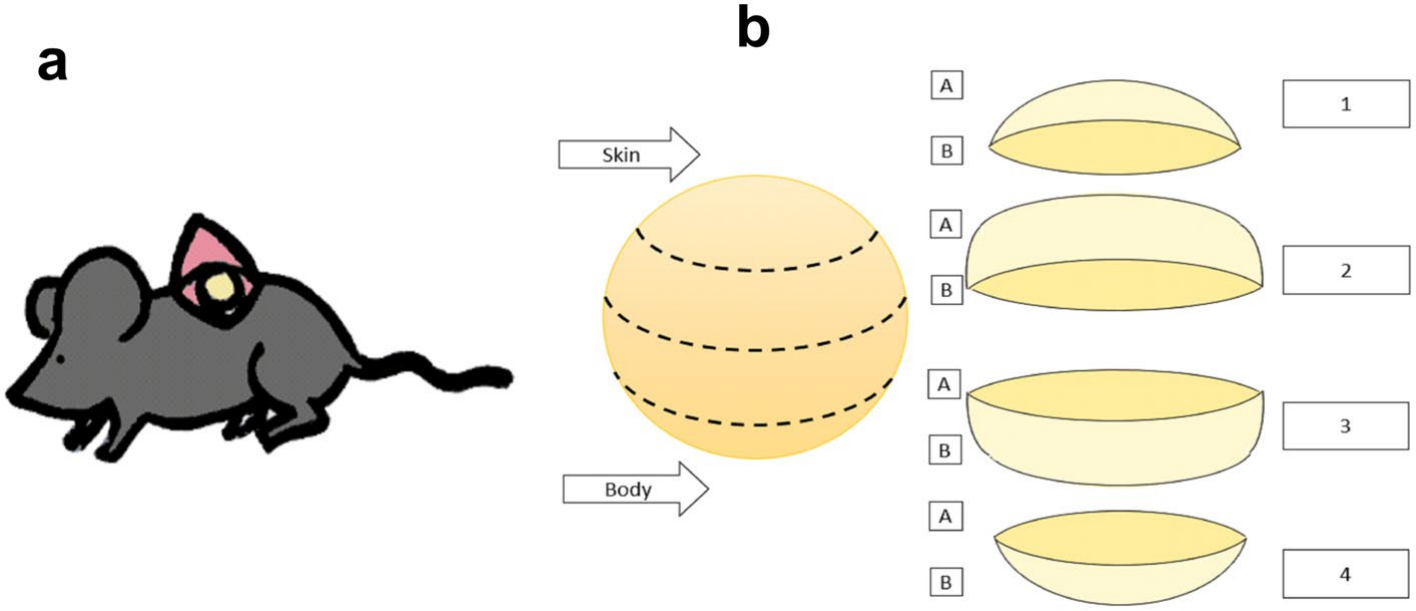
Procedure for tumor collection and segmentation for the study of the tumor. (**a**) Representation of the tumor implantation site at subcutaneous level in interescapular region. (**b**) Tumor segmentation scheme to define the tumor regions from which tissue sections were generated.

After anesthesia with ketamine (100 mg/kg) and xylazine (5 mg/kg), animals were perfused with 4% paraformaldehyde intracardially for complete fixation of the tissues prior to removal of the tumor masses. Tumors were progressively dehydrated in three sucrose solutions (10%, 20%, and 30%) for 10 min for each dilution. Once dehydrated, they were cut into four segments called 1, 2, 3 and 4, "Introduction" section being the tumor region in contact with the skin and 4, the one in contact with the body of the mouse. For each region, cuts were obtained from both sides available, named A and B for upper and lower, respectively (Fig. 5).

Tumor portions were placed in 24-well plates individually included in OCT Tissue Plus® compound for cryopreservation at –75 °C. Eight 10-µm cuts were made from each tumor region, four from each of its sides (side 1 and 2) (Fig. 5). Mice spleen cuts were used as macrophage staining control. The cuts were fixed on electrocharged slides and stored at − 20 °C until immunostaining and reading by immunofluorescence.

### Antibodies

An indirect immunofluorescence technique was standardized for the determination of intratumoral macrophage populations. Rat anti-mouse CD68 (NBP2-33337 Novusbio®) was used for the detection of total macrophages and revealed with donkey anti rat IgG antibody Alexa Fluor 488® (Jackson Immunoresearch). For the M1 subpopulation, monoclonal rabbit anti-mouse iNOS antibody (NB300-605 Novusbio®) and donkey anti-rabbit IgG Alexa Fluor 647® antibody (Jackson Immunoresearch®) were used. For the M2 phenotype, monoclonal goat anti-mouse antibody CD206 (AF2535 Novusbio®) was used, followed by donkey anti-Goat IgG Cy3 antibody (Jackson Immunoresearch®).

### Immunofluorescence staining analysis

Samples were placed at room temperature for 10 min, subsequently rehydrated in PBS 1X for 10 min, and incubated with blocking solution Immunodetector protein blocker/antibody diluent (BIOSB®) with 5% Donkey Normal Serum (Jackson Immunoresearch®) for 30 min at room temperature. Afterwards, three washes were carried out with Immunodna washer 1X (BIOSB®) solution and incubated with the primary antibody diluted in the Immunodetector protein blocker/antibody diluent solution with 1% DNS and 0.3% Triton X-100 detergent (Sigma-Aldrich) at 4 °C for 18–24 h. After three washes, a secondary antibody was added to the incubation solution and samples were incubated for 2 h at room temperature in the dark.

Staining was revealed with 1 µg/ml solution of diamino phenyl indole (DAPI) (Sigma-Aldrich®) for 15 min in the dark. After three final washes, samples were mounted on coverslips with DAKO Fluorescence Mounting Medium (Agilent®). Controls were slides with tumor tissue cuts which received all processing and buffers in the absence of primary antibody. Slides were read in a Molecular Device® Imagexpress® high sensibility multiplexed immunofluorescence microscope. For each tissue section, 12–16 reading fields were analyzed and images were processed by Metaxpress® program to quantify the macrophage populations (CD68 +) as well as the presence of iNOS for the M1 (CD68 + iNOS +) profile and CD206 for M2 (CD68 + CD206 +).

### Statistical analysis

Data analysis was performed with the IBM SPSS Statistics V20® software using the ANOVA test and comparison by Tukey’s test as a parametric test and Wilcoxon test for non-parametric variables. For ratio comparisons student-t test was carried out. For tumor implantation, Pearson´s Chi-Square test was performed, values of *P* < 0.05 were considered as statistically significant. Graphs were made with Graph pad Prism 8® software. Sample size calculation was made with G*Power 3.1.9.7 software.

### Ethics statement

This study was approved by the Institutional Sub-Committee for the care and use of Experimental Animals (CICUAE) of the Faculty of Veterinary Medicine and Zootechnics, UNAM (protocol number SICUAE DC-2019/2-4).

## Data Availability

The datasets used and/or analyzed during the current study are available from the corresponding author on reasonable request.
